# Mount Vernon Hospital

**Published:** 1902-07-12

**Authors:** 


### AROUND THE HOSPITALS.

### MOUNT VERNON HOSPITAL.

### THE YEAR'S WORK.

As already stated in these columns, the year 1901 was one of marked progress in the affairs of the hospital now to be known as "The Mount Vernon Hospital for Consumption, Hampstead and Northwood." Apart, however, from the great undertakings alluded to by Mr. B. A. Lyon, chairman, at the annual meeting in March, the ordinary routine work was greater than ever before. As a result of the increased accommodation provided at the close of 1900, a larger number of in-patients were admitted, viz., 578 instead of 493 as in the previous year. In the out-patients' department 4,699 were treated, 3,839 being the number in 1900. These numbers include the patients treated both at Hampstead and in Fitzroy Square. A new and larger outpatient department at Hampstead is needed. A daily average of a fraction over 98 patients is recorded in the medical report, 528 of the 578 being admitted for pulmonary tuberculosis. The waiting period has left its mark in the increased severity of the cases, and more beds are urgently needed. Thirty-nine patients are in the open air day and night, and many of them have appreciated the change from ward to balcony. Neither balconies nor wards are artificially heated. The treatment is in some cases carried on at home, and an example is given of a man who slept under a temporary shelter on the projecting part of the roof below his window. Protracted treatment is recommended in all possible cases, at sanatoria or elsewhere, as the maximum of three months is inadequate. Too much stress, the doctors say, cannot be laid on the value of the hospital as a means of preventing the spread of consumption by teaching clean habits. Each patient is now provided with a Dettweiler's sputum flask during the day, and these are thoroughly disinfected at night to be again ready for use. Paper handkerchiefs are used and burnt daily. A steam disinfector has been provided, in which all personal and bed clothing is rendered innocuous before being sent to the laundry. Annual subscriptions and donations have steadily increased during the past few years. The ordinary income in 1901 was £7,894, and the ordinary expenditure was £8,262. Church collections amounted to £102, and patients' payments to £4. King Edward's Fund gave £1,000 and a special donation of £50; increased grants were also received from the Sunday and Saturday Funds, the former giving £458, and the latter £190, with a special grant of £105. There was a legacy of £2,835. The gift of over £100,000, which has enabled the committee to buy a site for a convalescent home, has been already alluded to in THE HOSPITAL; a prettily wooded and sheltered park of 60 acres, some 16 miles from town, has been acquired, and as a result of competition, the designs of Mr. Frederick Wheeler, F.R.I.B.A., of 6 Staple Inn, have been accepted. There will be accommodation for 50 male and 50 female patients. In response to a special appeal, £9,700 has been received towards the completion of the Hampstead Hospital; the additional 40 beds are greatly needed, as 250 approved applicants are now awaiting admission. A nurses' home is also required, and when funds permit it is intended to build one in the grounds. The architects are preparing plans. Miss Whitton, the matron, is leaving after four years' useful work, and Miss Measures, late matron of Gravesend Hospital, has been appointed to succeed her.

